# Influence of Food Type on Human Psychological–Behavioral Responses and Crime Reduction

**DOI:** 10.3390/nu15173715

**Published:** 2023-08-25

**Authors:** Masoud Heidari, Yalda Khodadadi Jokar, Shirin Madani, Sharifeh Shahi, Mohammad Sharif Shahi, Mohammad Goli

**Affiliations:** 1 Department of Humanities and Law, Isfahan (Khorasgan) Branch, Islamic Azad University, Isfahan 81551-39998, Iran; masoud_heidari2@yahoo.com (M.H.); dr.shahi@khuisf.ac.ir (M.S.S.); 2 Department of Food Science and Technology, Laser and Biophotonics in Biotechnologies Research Center, Isfahan (Khorasgan) Branch, Islamic Azad University, Isfahan 81551-39998, Iran; 3 Department of Medical Engineering, Laser and Biophotonics in Biotechnologies Research Center, Isfahan (Khorasgan) Branch, Islamic Azad University, Isfahan 81551-39998, Iran

**Keywords:** food choice, food insecurity, mood, romantic relationship, crime, gut–brain axis, Halal–Tayyib foods

## Abstract

The purpose of this narrative review is to emphasize the importance of food consumption and meal selection on mental health and brain function, including psychological and behavioral reactions such as mood, loving relationships, violence, and criminal activity. Additionally, by being aware of the link between food and mental health, the community can be encouraged to make informed food choices in order to avoid unfavorable outcomes like criminality. Food behaviors are shifting significantly over the world. There are also significant changes in mood, sadness, happiness, and violence, as well as the spread of the variety and severity of mental diseases that lead to violent acts. Food intake and meal selection have evolved over the last ten years as the variety and accessibility of food options have become easier and more diverse. These modifications might have both beneficial and bad consequences. This article examines the relationship between food intake and its impact on marital satisfaction. The goal of this review is to support or refute the claim that food influences mood, love, or criminal behavior, or vice versa. Various diets can have an impact on one’s mental health and brain, influencing psychological reactions and behavioral responses such as mood, loving relationships, violence, and even criminal activity. Food insecurity has been demonstrated in various studies to have a negative impact on health and psychological well-being, leading to despair, loss of happiness, marital conflict, and violence. For example, herbal extracts and flavonoids have the potential to improve gut microbiota and treat mood disorders. Understanding how the gut–brain axis communicates might help guide interventions for mood and cognitive function. Since the root of most diseases and behaviors is significantly related to the type of food consumed, this research addresses this issue in order to reduce the cost of treatment and prevention of crime and delinquency at the community level by consciously choosing the food consumed by the society. In other words, prevention is always better than cure.

## 1. Introduction

Choosing healthy foods like vegetables and fruits improves mood and mental wellness. Fruit, vegetable, salad, and dairy product consumption has increased during the last decade [1]. On the other hand, people of all ages now have more access to and consume more fast food due to the growth of fast food restaurants in public spaces, including workplaces and neighborhoods [2]. Several academics have examined and reported on consumers’ perceptions and feelings about the impact of food choices on their happiness and mood shifts. Common human experiences include changing food preferences or choices in response to our transient psychological states, such as reaching for “comfort foods” in times of sadness or experiencing changes in hunger in response to stress. Furthermore, the links between nutrition and long-term mental disease are complicated by difficulties in maintaining a balanced diet. These disproportionate hurdles, which include economic and environmental health indicators as well as the appetite-related side effects of psychiatric medications, have an impact on people who suffer from mental disorders [3]. If there is proof that dietary changes have a positive impact on depressive symptoms, resources may be motivated to prevent and promote healthy living behaviors. Regarding the expenditures connected with mental illness, public health prevention that can result in a lower cost burden for society should be of importance [4]. A sedentary lifestyle, smoking, substance use, and unhealthy eating have all been linked to an increased risk of depression. These associations are strengthened when these lifestyle factors are combined [5].

The psychological factors influencing food choices have been linked to both short-term and long-term mood states, and it is known that dietary intake of particular nutrients can affect the biological processes underlying cognition, emotion, and behavior. Numerous studies have found that meal experiences have an impact on mood in both positive and negative ways [6]. Because of this, it can be challenging to pinpoint the beginning and end of the temporal food and mood cycle, though it has been suggested that some meals may do so [7]. According to studies, gender and personality are major factors in spicy food consumption. Spicy food preferences have been connected to greater levels of anger [8], and ingestion of chili peppers is associated with strength, daring, and masculine personality qualities in some cultural situations. According to a previous study, eating hot peppers is a thrill-seeking behavior with a high social component among American college students. High sensation-seeking and a preference for spicy meals have a substantial and positive association [9]. There is the possibility of receiving rewards like cash, sex, and social standing, which is consistent with historical accounts that claim eating spicy food was a sign of having a good social standing. Food insecurity has been linked to worse reading and arithmetic scores, as well as more internal and external problem behaviors [9]. While arithmetic, reading, and social skills have received a lot of attention, there has been less research on the influence of food insecurity on executive functioning. Executive functioning is significant because it is related to general cognitive processes and reasoning [10]. Executive functions collaborate to govern and coordinate cognition, emotion, and behavior, allowing a child to learn in school [11]. In terms of psychological qualities, one study found a link between a healthy diet and intellectually demanding work, low job demand, and strong job control in women [12]. Natural products’ therapeutic qualities may be due to their altering the microbiota–gut–brain axis. Flavonoids may have neuroprotective effects, counteract reactive oxygen species, and improve brain function and host behavior. Herbal extracts and substances in mood disorders can regulate gut microbiome structure and enhance depression-related behaviors [13,14].

One hypothesis suggests that food types, nutritional impact, and psychological–behavioral responses are linked to reducing crime. Nutritional impact plays a crucial role in brain function and neurotransmitter production, influencing behavior and mood. A balanced diet incorporating foods rich in micronutrients, like fish, is associated with lower aggressive behavior rates. High-fat and -sugar diets can affect brain neural pathways, potentially leading to withdrawal symptoms and behavioral changes. Food insecurity, especially during childhood, increases the risk of poverty and unemployment, leading to increased crime. Another study on the issue of work conditions and food choice coping strategies of employed parents found that working conditions are very effective in choosing between nutritious meals and fast foods, particularly in working mothers. As a result, it can have a significant impact on the family’s mental health. Meal preparation time has been reduced. Families are consuming more food and meals away from home, particularly fast foods and take-out meals [15]. Meal skipping and the usage of convenience dinners at home have both increased. There is evidence that meals made outside the home are less nutrient-dense than those prepared within. Negative work conditions including low employment status, poor working environment, heavy workloads, high work expectations, and a lack of control at work have all been associated with less healthy diets [16]. According to Devine et al. [17], excessive work expectations, long work hours, and high workplace stress have all been linked to weight gain and obesity.

## 2. Background

This narrative review posits that food consumption and meal selection significantly impact mental health and brain function, including psychological and behavioral reactions like mood, loving relationships, violence, and criminal activity (Table 1). This review highlights the need for further investigation due to the shift in food behaviors worldwide, changes in mood, mental diseases, and the spread of violent acts. The increased accessibility and variety of food options over the last decade may have both positive and negative consequences on mental health and behavior. The negative impact of food insecurity on health and psychological well-being, such as despair, loss of happiness, marital conflict, and violence, highlights the importance of understanding the relationship between food and mental health. Investigating how different dietary patterns and food availability influence these outcomes can provide valuable insights. The review emphasizes the importance of understanding the relationship between food consumption and mental health, behavior, and societal outcomes. The use of herbal extracts and flavonoids as potential interventions for improving gut microbiota and treating mood disorders supports the need for more information. Understanding the gut–brain axis communication and how dietary components influence this communication can guide interventions for mood and cognitive function. Addressing this issue can reduce treatment costs and prevent crime and delinquency at the community level. Preventive measures through conscious food choices are crucial. Further investigation in this field is needed to develop interventions that promote positive mental health outcomes and potentially prevent negative behavioral outcomes, such as criminal activity.

## 3. Methods

**Figure 1:**
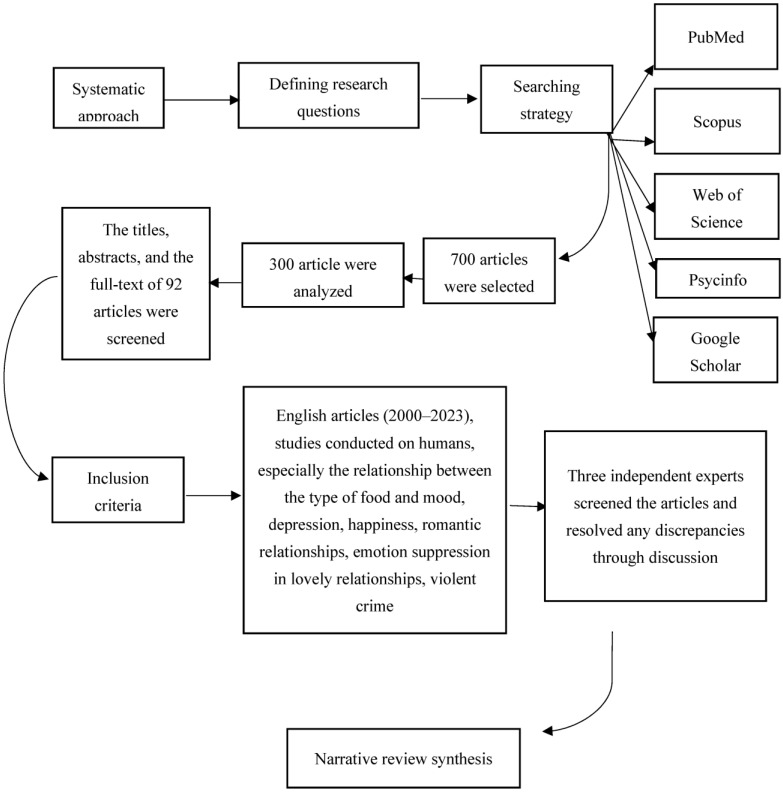
Schematic diagrams of the steps taken to conduct this narrative review.

In order to ensure transparency and rigor in this narrative review, the researchers followed a systematic approach to identify and collect relevant articles. They began by defining their research question and developing a search strategy that included specific search terms and databases. The search terms used in this study included food choice; food insecurity; mood; romantic relationship; and crime. They were searched for in the following databases: PubMed, Scopus, Web of Science, Psycinfo, and Google Scholar. After conducting the search, the researchers used a systematic approach to screen the articles and select those that met their inclusion criteria. To ensure the relevance of the retrieved articles to their review’s research question, articles that did not meet our inclusion criteria were excluded. The researchers employed a rigorous screening process. Initially, the search yielded 700 articles. After screening the articles, 300 were analyzed based on the exact present research question by the researchers, as well as a panel of experts (Figure 1). Finally, after removing duplicates, they screened the titles, abstracts, and the full text of 92 articles for relevance to their research question for eligibility. To ensure that all relevant studies were identified, the researchers applied the following inclusion criteria: articles published in English (2000–2023), studies conducted on humans, especially the relationship between the type of food and mood, depression, happiness, romantic relationships, emotion suppression in romantic relationships, and violent crime. For this purpose, in addition to researchers, three independent experts screened the articles and resolved any discrepancies through discussion. The researchers excluded papers that were not pertinent to their study issue or were rejected by their panel of experts, such as articles focusing on animal studies as well as previously listed non-targeted topics, or those published in languages other than English and before 2000. 

## 4. Results and Discussion

### 4.1. Food and Mood

**Figure 2:**
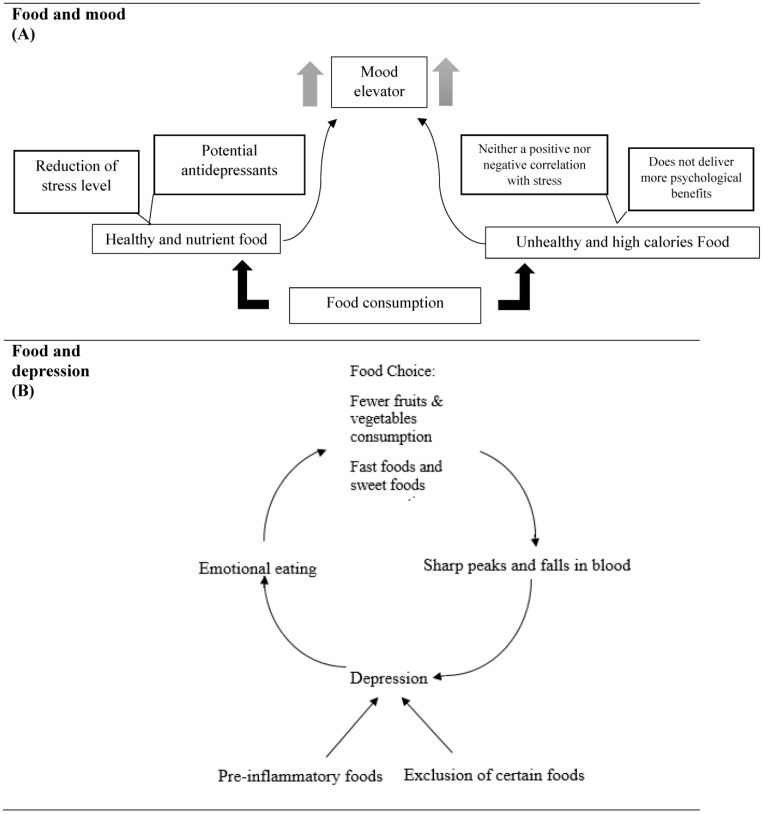

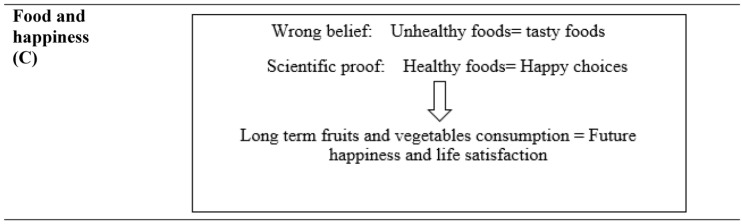
Schematic diagrams of the relationship between mood (**A**), depression (**B**), and happiness (**C**) and the type of food.

Contrary to the widely held view that foods heavy in fat, sugar, or calories taste better and make people happier, the idea that eating wholesome meals increases happiness and well-being has been put forth. Eaters naturally associate “unhealthy = tasty” and believe that sweets and chocolate are better mood enhancers than fruits and vegetables [18]. The anticipated enjoyment of eating must therefore be weighed against the likely problems and costs that may come from consuming such harmful and inadequately nutritious foods [19]. Numerous studies have demonstrated that feeling stressed out and depressed might lead to certain people overeating unhealthy meals (emotional eating) [20]. The role of unhealthy foods in improving mood status after a depressive mood episode was studied, and it was found that this impact was not present while eating in a controlled manner and with a normal mood (Table 1 and Figure 2A). These foods also have a weaker mood-elevating effect as compared to regular or healthy foods [21]. As a result, while some people believe that eating unhealthy foods provides more satisfaction and psychosomatic benefits, eating such foods does not deliver more psychological benefits than eating other foods. This misunderstanding may also be attributable to the fact that the majority of studies that examine how eating impacts mood, psychological status, mental health, and well-being primarily depend on retrospective evaluations such as meal frequency questionnaires [20] and written food journals. Self-report accuracy has a detrimental impact on whether the average intake depends on participants’ ability to accurately recall their eating episodes, which could lead to underreporting of food intake, especially of unhealthy snacks [22]. University students’ stress levels were shown to be reduced by eating fruits and vegetables, especially if they had a high body mass index (BMI), whereas chocolate and sweets had neither a positive nor negative correlation with stress. Healthy eating significantly reduced stress and depressive symptoms in students, regardless of gender or BMI [23]. Some other foods have also been attributed to better moods. For young men who do not have depression, walnuts, for instance, appear to elevate mood and ease tension or anxiety. The food’s strong antioxidant content, which includes folate, vitamin E, and omega 3 fatty acids with neuroprotective properties, is credited with this impact. Proteins high in tryptophan have been suggested as potential antidepressants [25]. Tryptophan is a crucial amino acid required for several metabolic processes, such as adult nitrogen balance, as well as for healthy growth in young children and newborns. It is used by the body as a precursor for the production of serotonin, which is believed to promote sound sleep and a cheerful mood [26].

This review suggests that the common belief that unhealthy foods taste better and make people happier may not be entirely accurate. Studies show that wholesome meals can increase happiness and well-being, while unhealthy foods may have a weaker mood-elevating effect. Healthy eating can reduce stress and depressive symptoms in students, regardless of gender or BMI. Some foods, like walnuts, have been linked to better moods and potential antidepressants. However, self-reporting on food intake may impact conclusions. Overall, healthy eating can have significant benefits for mental health and well-being (Figure 2A).

### 4.2. Effect of Different Foods on Mood

#### 4.2.1. Chocolate and the Other Sweets

Studies show that eating sweets and other sugary meals lifts mood, with women being more affected than males. According to Meier et al. [60], chocolate frequently evokes happy feelings and eases tension. This has a significant effect on mood. Chocolate is a popular choice as an anti-depressant because it contains psychoactive chemicals that target the opioid receptors in the central nervous system (e.g., anandamides), which act at the same site in the brain as cannabis, as well as tyramine and phenyl ethylamine, which act similarly to amphetamine [61]. This is in addition to theobromine and caffeine, which are also present and are known to have stimulating qualities. Due to its outstanding flavor and texture when taken, it causes chocolate cravings [62]. According to research, consuming high-cacao breakfast foods like high-cacao chocolate or biscuits makes people feel satisfied and less inclined to consume the rest of the morning. Anxiety has been linked more to excessive chocolate consumption than to moderate-to-low intake over time. After consuming chocolate, some weight-loss program participants feel horrible about themselves. Regular moderate-to-low consumption, however, has been linked to improved mood and enhanced happiness [63].

Studies show that sweets and sugary meals can boost mood, with women being more affected than men. Chocolate, a popular anti-depressant, has psychoactive chemicals targeting opioid receptors in the central nervous system. However, excessive chocolate consumption has been linked to more anxiety than moderate-to-low intake. Regular moderate-to-low consumption improves mood and happiness. Consuming high-cacao breakfast foods can make people feel satisfied, but it is crucial to consume chocolate in moderation to avoid negative consequences like anxiety and guilt.

#### 4.2.2. Caffeine

Caffeine is seen as a national drug that has the potential to impact daily mood and mental health. Its function as a central nervous system stimulant might momentarily increase activity. In chronic caffeine users, temporary caffeine shortage causes withdrawal symptoms. Caffeine consumption in the morning has been shown to dramatically lessen morning anxiety and improve performance [64,65]. It has been proven to significantly reduce fatigue and increase alertness. Consuming caffeine at various times and for varying lengths of time can drastically affect mood, enhance performance, and modify behavior. It has been suggested that regular coffee consumers are less likely to experience depression [66]. However, drinking too much caffeine can have a number of negative side effects, including insomnia, headaches, uneasiness, and stress [67]. James and Gregg recommended decreasing caffeine intake because they did not discover any appreciable benefits of caffeine on mood elevation. Researchers also advised men and women 45 years of age and older to consume less caffeine because this age group was more affected by caffeine’s ability to cause stress and worry [68]. However, because of the harmful side effects of larger levels, it is advisable to moderate caffeine consumption [69]. 

Caffeine, a national drug, can impact mood and mental health by stimulating the central nervous system and increasing activity, performance, and alertness. Regular coffee consumption may reduce depression. However, excessive caffeine consumption can cause negative side effects like insomnia, headaches, and stress. Research advises men and women aged 45 and older to consume less caffeine. Moderation is crucial, but excessive consumption should be moderated to avoid negative effects.

#### 4.2.3. Vegetables and Fruits

Because of their high nutritional value, eating more fruits and vegetables has also been linked to enhanced mood, cognitive function, and psychologic health in women. The daily recommended intake of vitamins and minerals for preserving good health and preventing disease is ensured by consuming a range of fruits and vegetables. It has been demonstrated that young people who frequently eat a variety of fruits and vegetables with a diversity of colors are happier, more imaginative, and more inquisitive [24]. Consumption of flavonoids, which are present in fruits and vegetables, may lower the chance of developing depression, according to epidemiologic research. Flavonoids enhance cognitive processes that are connected to executive function, which reduces depressive cognitive processes and elevates mood [19].

Consuming fruits and vegetables is linked to improved mood, cognitive function, and psychological health in women. These fruits and vegetables are high in nutritional value and provide essential vitamins and minerals. Consuming a diverse diet with fruits and vegetables can boost happiness, imagination, and inquisitiveness. Flavonoids in fruits and vegetables may reduce depression risk.

#### 4.2.4. Water

Mood is eventually impacted by inadequate water intake. Women who do not drink enough water were found to be more likely to be depressed, confused, tense, and angry. However, these mood-related symptoms had a positive or negative impact on how much water was taken during the day. Fluid deprivation is associated with dehydration, which results in fatigue, consciousness, decreased activity, decreased production of saliva, and poor sleep quality [70]. Armstrong et al. discovered a considerable correlation between dehydration and mood. They added that overall water consumption had an impact on both fatigue and intense activity. Dehydration in men has been linked to low energy, weariness, tension/anxiety, depressive symptoms, and poor memory [71]. 

Inadequate water intake can impact mood, with women experiencing depression, confusion, tension, and anger. Dehydration leads to fatigue, decreased activity, decreased saliva production, and poor sleep quality. Dehydration in men is linked to low energy, weariness, tension, depressive symptoms, and poor memory. Drinking enough water is crucial for both physical and mental well-being.

#### 4.2.5. Oilseeds and Seafood

It has been demonstrated that the omega-3 fatty acids eicosapentaenoic acid (EPA), docosahexaenoic acid (DHA), and alpha-linolenic acid (ALA), which are mostly found in oilseeds, all have positive effects on behavior, mood, neuroticism, and impulse control. These acids are also present in fish and other marine foods. According to Stahl et al. [72], they have positive effects on schizophrenia, drug abuse, major depressive disorder, bipolar disorder, and attention deficit disorder. Both EPA and DHA are essential for the growth and operation of the brain. These acids play a part in mood disorders and are connected to low blood levels in depression [73].

Omega-3 fatty acids, such as EPA, DHA, and ALA, have positive effects on behavior, mood, neuroticism, and impulse control. Consuming foods rich in these acids, like fish and oilseeds, can maintain good mental health and well-being. Deficiency has been linked to mood disorders and low depression levels.

#### 4.2.6. Spicy Foods

Despite the fact that consuming foods like hot peppers that contain capsaicin can cause discomfort, irritability, and even agony, many people all over the world, especially men, enjoy doing so. Sociologists have noted that the capacity of spicy food to elicit these unpleasant physiological responses has led to an association between these meals and qualities associated with masculinity in various cultural contexts around the globe [74]. The proposed relationship between a preference for spicy food and endogenous testosterone, a hormone frequently associated with stereotypically masculine preferences and behavior, was evaluated in one study. Numerous factors, including genetic, psychological, physiological, and social ones, affect people’s preferences for and consumption of foods containing capsaicin [75]. According to studies, personality types and gender are important factors in the consumption of spicy meals. In some cultural contexts, eating chili peppers is linked to traits of power, bravery, and masculinity [8]. Spicy food preferences have also been linked to higher levels of trait anger. Eating hot peppers is occasionally a thrill-seeking behavior with a social component in American college students, as described by Byrnes and Hyes [9]. The preference for hot meals and strong sensation-seeking are significantly and positively correlated [9]. It is consistent with historical stories that consuming spicy meals served as a mark of high social standing, and that a person’s love for spicy foods is associated with responsibility for rewards such as money, sex, and social prestige. Last but not least, persons who are portrayed as favoring spicy foods may be angrier than those who prefer sour, sweet, or bitter meals. It has been proposed that dietary intake is relevant to hormonally related behaviors, especially those affected by testosterone. Several aspects of the liking for capsaicin have been repeatedly linked to this hormone, including social dominance and aggression [76], novelty- and sensation-seeking, and daring behavior [77]. On the other hand, decreased testosterone levels have been linked to other behaviors incongruous with using capsaicin, such as a lethargic, drowsy or depressed mood [78]. 

Consuming spicy foods containing capsaicin can cause discomfort, irritability, and agony, but many people, especially men, enjoy them. Studies show that personality types and gender play a significant role in the consumption of spicy meals. Consuming chili peppers is linked to traits of power, bravery, and masculinity. Spicy food preferences are also linked to higher levels of anger and sensation-seeking behavior. Hormonally related behaviors, such as testosterone, are also associated with dietary intake, including social dominance, aggression, novelty, sensation seeking, and daring behavior.

Despite the widely held perception that unhealthy, high-fat, and high-sugar foods taste better and make people happier, evidence supports the idea that eating healthy meals enhances happiness and well-being. Negative mood outcomes have been associated with emotional eating, which entails overindulging in unhealthy foods in response to stress and melancholy. Eating unhealthy foods does not provide more psychological benefits than eating other foods, since they do not have as strong of an influence on mood elevation as regular or healthy foods. Consuming caffeine has been shown to increase performance and lessen morning worry and exhaustion, but too much of it can have adverse consequences like stress, insomnia, migraines, and anxiety. By improving executive function-related cognitive processes, lowering depressive cognitive processes, and boosting mood, flavonoids—which are found in fruits and vegetables—may reduce the risk of depression. Lack of water consumption can cause sadness, disorientation, stress, and rage, especially in women. Dehydration is linked to weariness, decreased activity, and poor sleep quality. Spicy food preferences have been connected to gender, personality types, and in some cultural contexts, qualities of strength, bravery, and masculinity. It is also seen as a thrill-seeking activity with a social component. Consuming spicy foods has been linked to attributes like testosterone-influenced social dominance, aggression, novelty and sensation-seeking, and adventurous behavior.

### 4.3. Food and Depression

The most prevalent psychiatric condition that influences mood, emotion, and behavior is depression (Table 1 and Figure 2B). Men and women of diverse ages, from many backgrounds and cultures, are affected. Emotional eating can result from depression and have a significant impact on diet decisions. Due to technology developments, many jobs in today’s society are less physically demanding, but the psychological stress has increased. A person with a mental illness may struggle to manage their daily affairs, including their family and job, and seeking treatment for a mental illness may be complicated by feelings of guilt and shame [5]. Existing research on lifestyle habits in treating depression has been summarized in a narrative review. Three clinical suggestions were produced by that analysis. The first was that poor eating, smoking, an inactive lifestyle, and substance use were lifestyle variables that raised the risk of depression, and many of these factors occurring simultaneously strengthened the relationship [5]. The second clinical recommendation was supported by research showing that engaging in physical activity reduces symptoms of depression. The third advice was based on just a few studies, but it nevertheless showed the value of recommending diet changes and help for quitting smoking as a depression treatment [4]. Depressed people who participate in emotional eating consume more sweet foods, whereas depressed people who do not engage in emotional eating consume fewer fruits and vegetables; hence, sorrow and emotional eating both influence dietary choices and increase consumption of dangerous foods. Eating high-fat, high-sugar foods like sweets and fried food boosts cheerful mood via increasing activity in the reward centers of the brain [79]. Furthermore, there is emerging evidence that simply seeing visual pictures of comfort foods (high fat and high sugar) without eating enhances neuronal activity in brain reward regions [80]. 

Exclusion of certain foods from a person’s diet has been related to an increased risk of getting depression. Vegans have been found to have the highest rate of depression symptoms (28.4%), whereas omnivores had the lowest (16.2%). According to one study, the risk of depression increased dramatically with the number of food items eliminated, regardless of diet [29]. Certain vitamin deficits can cause depression or aggravate its severity. A clinical study showed a link between folate and neuropsychiatric disorders; most depressive people are deficient in folate, which lowers the efficiency of antidepressant drugs. Additionally, reduced fish consumption in depressed males may worsen sadness and decrease appetite [30]. Similarly, a lack of omega-3 sources such as fish and nuts might exacerbate anxiety and sadness. Using omega-3 supplements may help with mood, anxiety, and depression. Finally, eating can affect one’s mood, either favorably or unfavorably. Deficiencies in vitamin B complex, vitamin D, vitamin C, zinc, omega-3 fatty acids, and antioxidants, among others, can have a bad effect on mood and may eventually lead to depression. The neurotransmitters that control mood are balanced and controlled by these nutrients. Fruits, vegetables, and dairy products are excellent sources of these nutrients [27]. Anti-inflammatory foods can reduce symptoms and ward off mental illness, but diet can affect the body’s level of inflammation and may be linked to a higher risk of depression. One study discovered that a high consumption of pro-inflammatory meals raised the chance of developing depression, whereas a diet low in anti-inflammatory foods reduced the incidence of depression and its symptoms. Research from other countries that associated a diet high in pro-inflammatory foods to a greater prevalence of depression and anxiety supports these findings [31]. One study found that taking probiotics and prebiotics improves gut health, which reduces inflammation and hence results in fewer symptoms of mental illness [81]. Pro-inflammatory foods should be avoided before and during mental illness as a preventive measure. Clinical investigations have also demonstrated that refined carbs may have a causal effect on mood; healthy volunteers’ depressive symptoms are slightly worsened when they are experimentally exposed to high-glycemic-load diets in controlled environments.

Even while our moods can affect our food choices, there are likely mechanisms by which higher consumption of processed carbohydrates could enhance the risk of depression and anxiety, such as through frequent, sharp peaks and falls in blood sugar. In healthy individuals, glycemia and insulin demand after eating can be estimated by using the glycemic index and glycemic load measures [82]. Because of this, a high meal glycemic load and the resulting compensatory responses may cause plasma glucose to fall to levels that trigger the release of autonomic counter-regulatory hormones such as adrenaline, cortisol, growth hormone, and glucagon [83]. Human experimental research employing stepwise plasma glucose concentration reductions carried out under lab conditions using glucose perfusion have looked into the potential effects of this reaction on mood. These results demonstrated that these hormones could modify anxiety, irritation, and hunger. Recurrent hypoglycemia (low blood sugar) has also been connected to mental illnesses, according to observational studies [32].

Numerous studies on the connection between food and depression have shown that unhealthy eating, smoking, sedentary lifestyles, and substance abuse are lifestyle factors that increase the risk of depression. It has been demonstrated that changing one’s diet and engaging in physical activity can help treat depression. Depression can cause emotional eating, which can significantly influence diet choices and result in the ingestion of high-fat, high-sugar meals that improve mood by stimulating the reward centers of the brain. Vegans had the highest rate of depressive symptoms; however, eliminating specific foods from one’s diet has been linked to an increased risk of depression. Deficits in some vitamins can lead to depression or make it worse, and a lack of omega-3 sources like fish and nuts can make anxiety and melancholy worse. Additionally, refined carbohydrates may have a negative impact on mood, and high meal glycemic loads can cause plasma glucose to drop to levels that stimulate the release of hormones that control appetite, irritability, and anxiety in the body. As a result, it is critical to understand how nutrition might affect mental health and to work towards eating a balanced, nutrient-rich diet that includes probiotics and foods that reduce inflammation (Figure 2B).

### 4.4. Food and Happiness

Researchers, the media, and policymakers have categorized the detrimental aspects of eating behaviors, such as restricting particular meals, keeping track of calories, and dieting. Similar to this, health intervention initiatives such as primary prevention programs typically encourage consumer behavior to trade off anticipated enjoyment of pleasant and comfort meals for health benefits [33]. Restrictive eating patterns and diets, on the other hand, have been found in studies to be frequently ineffectual and even to increase the risk of long-term weight gain and disorders related to eating. A possible new viewpoint involves a change away from seeing food as just energy toward one that emphasizes happiness and human eating behavior [84]. Block [34] has argued for a paradigm change from “food as health” to “food as well-being” in this situation. The idea that “good” dietary choices, such as increasing fruit and vegetable consumption, provide advantages for both physical and mental health and may even be a long-term investment in future well-being is supported by a number of studies. For instance, Mujcic and Oswald [20] found that consuming fruit and vegetables over a two-year period predicted increases in happiness, life satisfaction, and well-being in a nationally representative panel survey of more than 12,000 Australian adults. Similar to this, the previous researcher demonstrated using lagged analyses that a diet including fruit and vegetables did not cause improvements in positive affect on the following day [85]. Furthermore, a cross-sectional study shows that eating fruits and vegetables is connected with increased happiness after controlling for demographic characteristics such as age, gender, and race [35]. The results provide credence to the idea that eating fruits and vegetables over a wide range of time periods has positive benefits on many well-being markers, such as happiness or overall life satisfaction. The notion that eating more fruits and vegetables and other healthy food options is associated with happiness and wellness contrasts sharply with the widely held belief that high-fat, high-sugar, or calorie-dense foods feel better and make us happy while we eat them. When it comes to food, people typically equate “unhealthy” and “tasty” and believe that chocolate is a greater mood booster than an apple [18]. Customers must measure the expected pleasure of dining against the health costs of eating unhealthy meals, according to this moment-to-moment well-being perspective [33]. Eating “unhealthy” comfort food had a mood-enhancing impact after a negative mood induction, although not to the same level as eating non-comfort or neutral food, according to one of the few studies that examined the effectiveness of comfort food in elevating mood [20]. Because of this, eating “unhealthy” foods may not be psychologically better than eating other foods, despite what some individuals may believe when they snack on “unhealthy” items like ice cream or chocolate.

Although many cultures communicate the impression that eating certain meals has a greater hedonic and mood-boosting effect, the current data suggest that this may not accurately represent real in-the-moment sensations. Despite the fact that people frequently have a spontaneous “un-healthy = tasty” intuition, indicating that a stronger effect of “unhealthy” food on boosting happiness is to be expected, the average level of induced eating happiness for sweets was the same as that for “healthy” food options like fruits or vegetables. This was also true for other clichéd “unhealthy” items like salty snacks and pastries, which did not increase happiness as much as was anticipated. Moreover, evaluations at the meal-type level are consistent with this idea, because supper had a similar “happy” characteristic to snacking, despite snacks having a generally positive effect. When compared to stereotypically “unhealthy” food choices, “healthy choices” appear to be “happy choices” as well, with a hedonic value at least equal to or even higher than unhealthy foods. Many people think that certain foods are more comfortable than the others are. Moreover, high-calorie foods are often associated with being consumed in reaction to negative emotional stress because they are thought to have rapid psychophysical benefits [86]. Comparing various meal types, however, did not support the idea that they varied in the level of comfort they offered; rather, eating generally resulted in appreciable mood elevations. When “good” food options like fruits and vegetables were compared to “unhealthy” food options like sweets, very comparable patterns emerge since both were, on average, associated with high levels of eating enjoyment and had a wide variety of experiences, from extremely negative to extremely positive (Table 1 and Figure 2C) [20].

This review addresses the connection between food and happiness and refutes the widespread notion that eating “unhealthy” foods with a lot of fat, sugar, or calories makes us happier. The authors contend that a paradigm shift is necessary from one that focuses on happiness and human eating behavior to one that views food as more than just energy. We reference a number of studies to show how making “good” dietary decisions, including boosting fruit and vegetable consumption, can benefit both physical and mental health. These decisions may even be a long-term investment in future well-being. The effects of restrictive eating habits and diets on long-term weight gain and eating disorders are also covered (Figure 2C).

### 4.5. Romantic Relationships and Food

**Figure 3:**
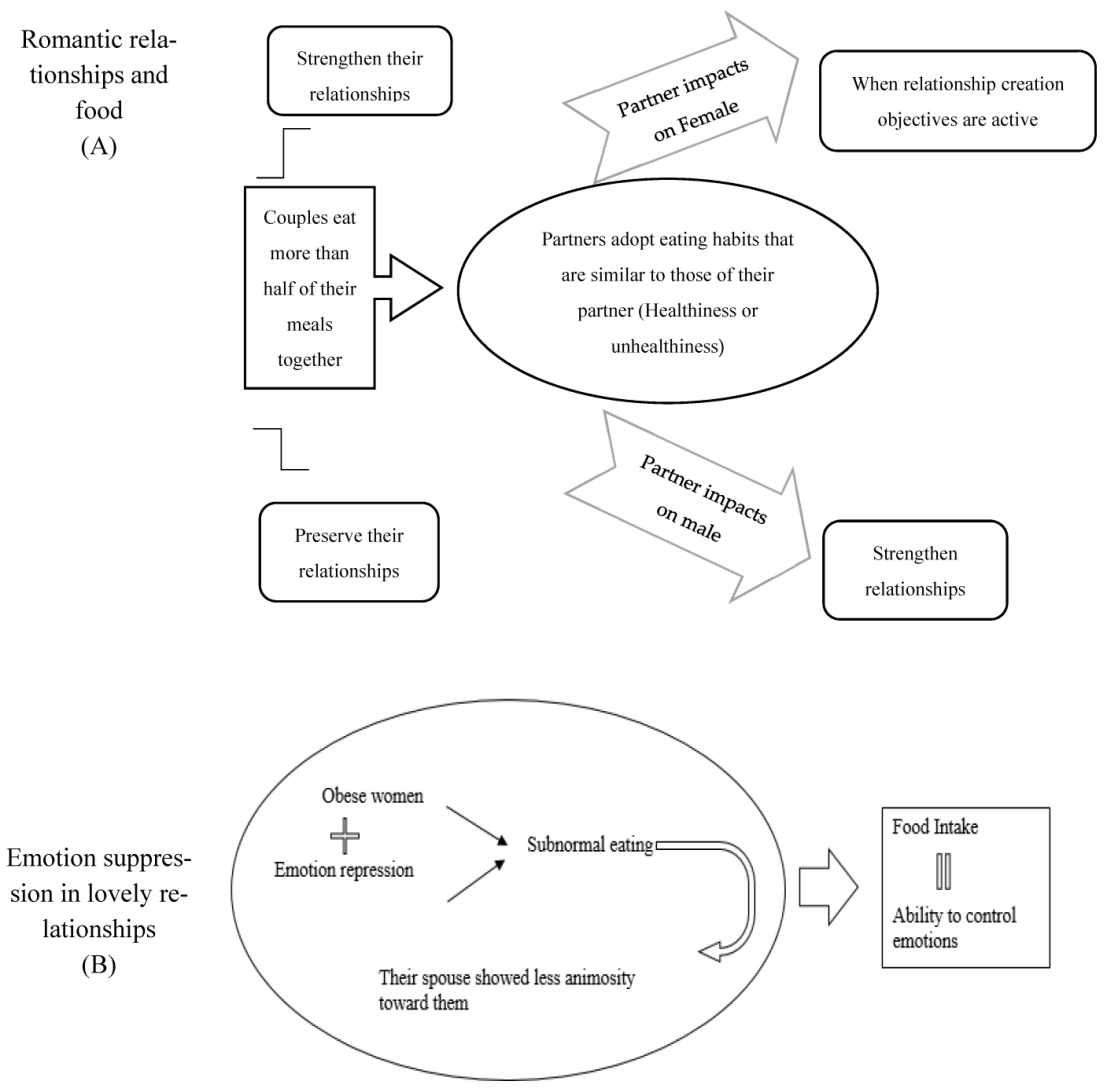
Schematic diagrams of the relationship between romantic relationships (**A**), emotion suppression (**B**), and food choice.

Interpersonal influence from friends, coworkers, and family frequently influences how people make decisions on their own (Table 1 and Figure 3A). These social influencers have significant effects on how people form attitudes, choose products, and identify with particular reference groups [36]. When it comes to having an impact on others, perhaps no source of influence has more power than the person you love. The special sharing of resources and ambitions in a romantic partnership means that people are frequently influenced by the preferences and beliefs of their partners. When making decisions, for example, people in romantic relationships may take into account the attitudes and opinions of their significant other [37]. Despite these significant findings, there has been surprisingly little research on how a romantic partner might affect one of the most frequent and routinely made collaborative decisions—choosing food. According to study [38], couples eat over half of their meals together, and eating together is a common practice for partners to both strengthen and preserve their relationships. Additionally, examining food preferences in romantic relationships may provide crucial insights into why people eat unhealthy foods and when conditions like obesity may develop [39]. Furthermore, in order to maintain harmony in a relationship or prevent conflict with a spouse, it is possible for individuals to adopt eating habits that are similar to those of their partner. The factors that could affect whether men or women are impacted by the dietary preferences of their romantic partners are also discussed. Hasford et al. [39] looked into how romantic partners affect dietary decisions. Regarding the relational incentives that are active at the time of consumption, they place particular emphasis to how males and females are affected by the opposite sex. Furthermore, in order to maintain harmony in a relationship or prevent conflict with a spouse, research indicated that, whereas male eating habits (i.e., healthiness/unhealthiness) have an impact on females when relationship creation objectives are active, female eating patterns have an impact on males when relationship maintenance motives are active. Additionally, they provided proof that beliefs of relational impact alter their observed effects. When dining with one’s boss (who possesses high levels of relational maintenance), the pattern of results was comparable to females (males) in relationship creation. Additionally, the study discovered that the association between motivation, gender, and food preference is modified by perceptions of relational influence [40].

This review discusses how romantic connections affect what people eat and how they choose to eat. According to the author, love partners significantly influence one another’s food preferences, and analyzing food preferences in romantic partnerships may offer vital insights into why people eat unhealthy foods and when diseases like obesity may manifest. According to a study included in this review, partners frequently practice eating together to both develop and preserve their relationships. Couples eat more than half of their meals together. The passage makes the suggestion that people might emulate their partner’s eating habits in an effort to keep things amicable or avoid arguments. This review also explores how, depending on the relational incentives that are in play at the time of consumption, the effects of food choices on males and females may vary. According to the research given in this review, male eating behaviors have an effect on females when relationship-creation goals are present, whereas male eating behaviors have an effect on females when relationship-maintenance goals are present. The study also found that perceptions of relational influence alter the relationship between motivation, gender, and food preference. In conclusion, this review presents scientific evidence to support the notion that romantic partners significantly influence each other’s food preferences and eating habits, and that investigating food preferences in romantic relationships may offer vital insights into why people eat unhealthy foods and when conditions like obesity may develop. According to this review, people may adopt their partner’s eating habits in an effort to keep things amicable or avoid conflict. It also contends that the effects of dietary preferences on men and women may vary depending on the relational incentives that are in play at the time of consumption (Figure 3A).

### 4.6. Emotion Suppression in Romantic Relationships

Many studies have been carried out on emotional eating during the last few decades (Table 1 and Figure 3B). Negative emotions and eating have a complicated association since numerous elements, including emotion intensity and individual traits, might influence this relationship [38]. Researchers are increasingly interested in unraveling the pathways connecting emotion to food consumption. They suggest that this association may be mediated by how people manage their emotions. Among the approaches for emotion control, emotion suppression has been identified as a critical role in emotion-induced eating. Emotion suppression is the suppression of emotion expression while emotionally aroused in order to conceal one’s emotional condition from others. This strategy emphasizes controlling one’s behavioral reactions to an emotional situation. Because emotion suppression requires effort, it has been linked to undesirable behavioral consequences such as smoking and drinking [28]. One study looked at the interaction between eating and suppressing emotions in the context of romantic relationships. Butler et al. [41] conducted a 7-day diary study on 91 heterosexual couples to assess eating behavior, emotion suppression, and joyful and negative feelings. Emotional restraint and food intake were found to be related in females. This connection was constrained by BMI. In fact, overweight/obese women ate more than normal on days when they reported having high levels of emotion suppression, whereas this link was not seen in non-overweight/non-obese women. However, no connection between the feelings males experienced in relationships, the suppression of emotions, and eating was discovered [41]. A dyadic effect was also evident in the results. The husband of an obese woman expressed less animosity toward them on days when the woman reported high levels of emotion repression. The spouse of non-obese/non-overweight women who repressed their emotions, on the other hand, felt more negatively about them [41].

Researchers found that both personal mood regulation and the emotion regulation of the other partner affect food consumption. It is believed that a person’s ability to control their emotions has a big influence on how food intake and mood are related. According to these results, it seems that watching one’s love partner suppress their emotions (and thinking that they are emotionally cold) may have caused one to eat more while feeling emotionally stimulated by the tense couple conversation. This is in line with other studies on couples and eating behaviors, which suggests that spouses’ perceptions of their partners may influence how they control their eating habits [42]. Three themes emerged from the use of thematic templates: (i) the social environment, which includes cultural and familial influences on food and mood; (ii) social economics, which includes time, money, and food security; and (iii) food nostalgia, which involves bringing back memories that have an impact on mood. These subjects brought to light the importance of social context, life experiences, attitudes, expectations, and values in the link between food and mood [43].

One social context theme is the effects of culture and family on eating and mood. Participants in this theme acknowledged that their youth, upbringing, family dynamics, and food culture were significant parts of the social environment surrounding food and its relationship to mood. According to Higgs and Thomas [44], there may be a societal influence on people’s eating behaviors. The environment and social context influence dietary habits, and these social standards may be seen as an adaptation to reward. The behaviors of the company at the table, as well as their culture and upbringing, set social norms that have an impact on food choices [45]. Frequently occurring social eating activities, such as preparing and sharing meals, singing, dancing, laughing, and discussion, improve relationships and general wellbeing. Social eating also results in the release of endorphins, which foster bonding [46]. Research on the social determinants of food intake and diet suggests that mood plays a significant role in this relationship. Social ties and relationships are strengthened when people eat meals together. Neurotransmitters and endorphins are released during social eating, which fosters comradery and positive sentiments. According to focus group results, social eaters have a better quality of life, are more active in their communities, and have a wider network of close friends [46].

A second theme is “social economics: timing, money, and food security.” Participants in this theme talked on how time constraints and other issues with food security, such as cost, affected their eating preferences and disposition. They said that preparation, organization, and planning had aided in their better food decisions, which had a positive impact on their mood. Their dietary decisions were influenced by their lack of access to food, their lack of free time, and their financial limitations, all of which had an impact on their mood and mental health. Food insecurity is defined as the insufficient availability of nourishing foods and is linked to stress and uncertainty brought on by not knowing where one will be able to find their next nourishing meal [47,48]. Lower socioeconomic groups are more likely to experience food insecurity, which is directly driven by growing food prices and affordability and indirectly by time pressures brought on by having to work longer hours to cover basic needs. Furthermore, food insecurity has a detrimental effect on one’s wellbeing and mental health. Chronic stress brought on by food insecurity can alter how the body metabolizes nutrients and how people respond to food, which can worsen physical and psychological outcomes [49]. Price, disposable income, food access, and availability are other important factors that influence food choices due to food insecurity. In communities with food insecurity, foods that are affordable, accessible, and readily available are usually processed, quick, or sweet, which has an unintended negative effect on residents’ mental health. According to Lee et al. [50], those who live in food-insecure areas are more likely to experience depression, anxiety, poor sleep, and low mood than people who live in food-secure areas. The third theme is food nostalgia, which explores how memories of food might influence mood. The focus of this theme’s discussions in the focus groups was on the role that memories and food had in creating both happy and depressing feelings. The passages that follow illustrate how participants’ ingestion of typical foods from their past helped them remember pleasant moments by invoking nostalgia and bringing back memories of loved ones who had passed away: food nostalgia is a novel and developing concept that lacks a clear definition and adequate research [51]. As opposed to the traditional definition of nostalgia, which implies ambivalent or negative emotions, an exploratory qualitative analysis of 104 explanations revealed that food nostalgia was associated with yearning, childhood, homesickness, rediscovery, special occasions, and substituting, as well as that food nostalgia is associated with positive emotions [52].

The relationship between unfavorable feelings and eating behavior is discussed in this review, as is the probable role of emotion suppression in emotional eating. In one study that examined the association between eating behavior and the suppression of emotions in the setting of romantic relationships, it was discovered that obese and overweight women ate more frequently than usual on days when they reported having high levels of emotion suppression. The relationship between males’ emotions and eating behavior, however, was not discovered. According to that study, individuals may have eaten more while feeling emotionally stimulated by the stressful marital dialogue as a result of witnessing their spouse hide their emotions. The relationship between food and mood is further highlighted in the literature by highlighting the significance of social context, life experiences, attitudes, expectations, and beliefs. The study’s participants admitted that their childhood, family dynamics, and food culture were important components of the social context surrounding food and its connection to mood. According to the paper, communal eating activities that involve preparing and sharing meals encourage happy emotions and close relationships. The study also revealed that social economic factors, such as time restraints, money constraints, and food insecurity, had an effect on eating preferences and mood. Participants stated that their mood and mental health were impacted by their lack of access to food, their lack of free time, and their financial situation. Food nostalgia, the third theme covered in this review, examines how the way we feel about food might be influenced by our memories of it. Participants in the study claimed that nostalgic memories of familiar foods from their past helped them recall happy experiences and evoke positive feelings. In general, this review sheds light on the intricate connection between emotions, eating habits, and social setting. We make the case that, particularly in the setting of romantic relationships, emotion suppression may play a significant role in emotion-induced eating. This review also emphasizes how social setting and life experiences play a significant role in determining one’s mood and eating preferences (Figure 3B).

### 4.7. Food Insecurity Effect on Violent Crime

**Figure 4:**
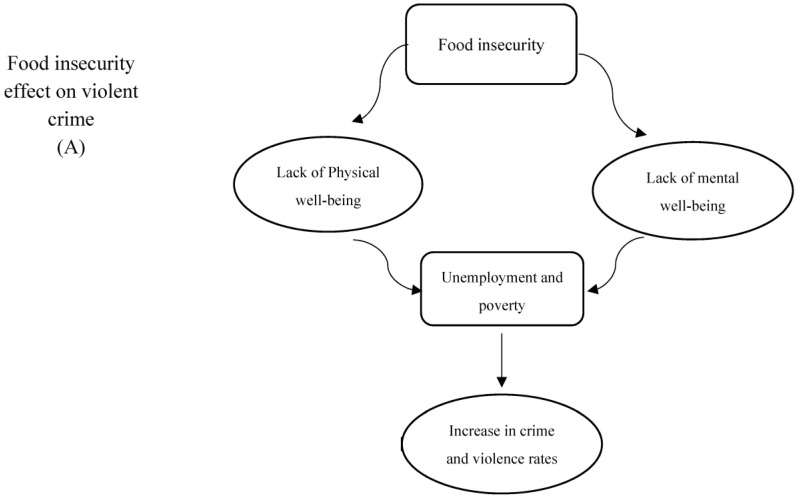

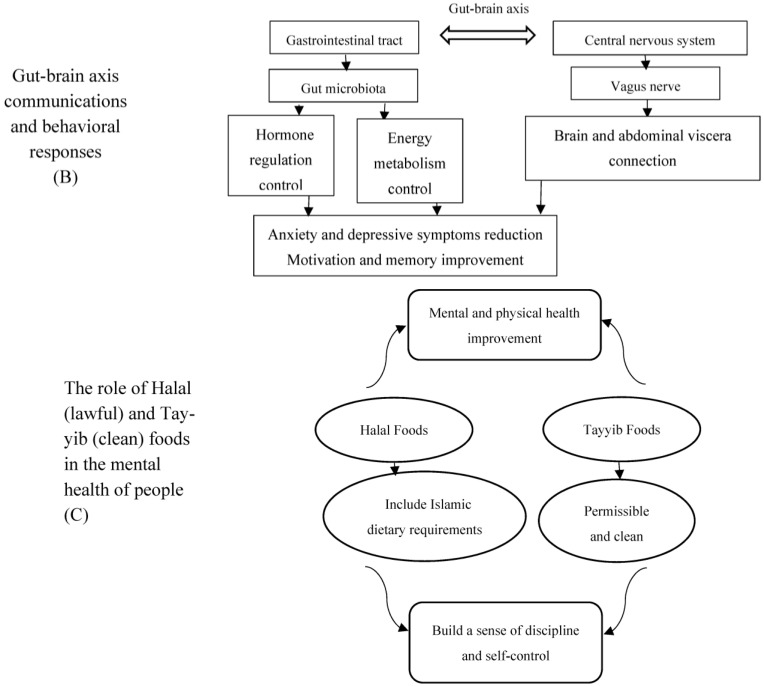
Schematic diagrams of the relationship between food insecurity’s effect on violent crime (**A**), Gut–brain axis communications and behavioral responses (**B**), and the role of Halal (lawful) and Tayyib (clean) foods in the mental health of people (**C**).

Violence crime and food insecurity are related (Table 1 and Figure 4A); however, the effects vary depending on the type of crime [53]. What are we becoming if it is true that we become what we consume? Nobody would deny that the growth in obesity is a result of our diet, but what we eat also affects how our brains are programmed, contributing to the buildup of body fat. Food gives our brains the energy they need to function as well as the building blocks for neurotransmitters that affect brain communication and significantly affect the environment in which the brain operates [54]. The brain only makes up 2% of body mass but uses up to 20% of the energy that is available. However, it appears that we have made considerable changes to current diets in a short amount of time, with little to no systematic evaluation of potential consequences on behavior or brain function. Not widely known is the fact that neither brain function nor behavioral results were taken into account while developing our nutritional adequacy criteria. Given this, it is possible that we significantly overestimated the influence of these physiological factors, which we should pay attention to at all costs [55]. It has been discovered that a lack of food has a detrimental effect on people’s physical and mental health. Brinkman [87] conducted research that showed food insecurity increases the likelihood of violence and civil strife in developing countries. Unfortunately, very little study has been undertaken in wealthy countries to investigate a possible link between food insecurity and violence. At the county level, Caughron [53] looked into a potential link between food insecurity and violent crime rates in the US. He also looked into how the degree to which independent variables like the county’s revenue and population affect the relationship between food insecurity and violent crime. Finally, the model breaks down violent crime into separate offenses and investigates how food insecurity affects crime rates on an individual basis. The results showed that, when other predictors of violent crime remain unchanged, a 1% increase in food insecurity results in a 12% increase in violent crime. Depending on the population and financial standing of the county, many factors influence how food insecurity affects crime rates. A review of the literature reveals that only one book specifically addresses the link between food insecurity and racial violence in the United States. Kent [88] performed a survey of juvenile delinquents to see if food-poor children were considerably more likely to engage in criminal activity than food-secure youngsters. According to the findings, food-needy children were no more likely to participate in criminal conduct than food-secure individuals. While Kent’s paper is a good beginning point for further investigation into the association between food insecurity and crime, the findings are only applicable to a tiny segment of the American population [88]. Previous researchers have investigated the extent to which food production losses may exacerbate violent conflict. They analyze three assessments of the severity of violent conflict from 1982 to 2011 using data from India [89]. The authors discovered that higher food production reduces the severity of violent conflict over time. They discover, however, that only the delayed effect of food production is significant at the 5% level. This finding appears fair, as a reduction in food production is unlikely to have a substantial impact until present food stockpiles begin to deplete [89]. The literature on food insecurity and factors that may affect violent crime rates provides compelling evidence that experiencing food insecurity, especially as a child, significantly increases the likelihood that an individual will later experience poverty and unemployment, which increases the likelihood that they will turn to crime. Food insecurity has also been connected to mental health issues in all 16 age groups. A rise in the rate of mental illness may result in dysfunctional or single-parent homes, as well as problems finding or maintaining a job and decreased income, all of which may contribute to crime [8]. The consumption of fish, a balanced diet high in micronutrients, and general excellent nutrition have all been linked to lower rates of violent behavior [55]. One study provided evidence to support this since the participants were unable to correctly determine the type of capsules they had been given. Here, unlike with alcohol, we have a strong influence on behavior that seems to operate without our consciousness. Because of this, if someone is unknowingly undernourished, individuals around them are unlikely to be aware of it and will likely mistake any incorrect behavior for personality flaws, etc. It is obvious that when more social interactions are susceptible to these hitherto unknown influences on human behavior, these difficulties will certainly drastically expand on a societal level. Without our knowledge, this might change socially acceptable behavioral norms [55]. How humans interact with food is probably influenced by a number of factors, including physiology, family, peer group, choice, economy, physical activity, and food distribution. It is intriguing to think about why we do not make better eating choices given the huge selection of nutritious foods that are readily available today. It has been claimed that excessive energy density leads to increased calorie intake, since inadequate satiety signals cannot make up for it [90]. According to Prentice [91], high-fat and high-sugar diets have the same impact on the brain’s neural pathways as addictive substances. When this is withdrawn, they display withdrawal symptoms similar to those caused by addictive substances. Studies involving drug users are progressively confirming these results. It is worth noting that national sugar consumption has reportedly increased sevenfold in just 200 years [55]. 

In this review, we discuss how the connection between hunger and violent crime differs based on the type of crime. This review also emphasizes how food affects how the brain works and how people behave, with a focus on how nutrition affects the neurotransmitters that control brain communication. According to this study, a lack of food has a negative impact on people’s physical and mental well-being and raises the risk of violence and civil unrest in developing nations. The literature on food insecurity and factors that may influence violent crime rates shows conclusively that having food insecurity, especially as a child, significantly raises the risk of later experiencing poverty and unemployment, which raises the risk of turning to crime. There is evidence that eating fish, eating a well-balanced diet rich in micronutrients, and generally eating well are all associated with lower incidences of aggressive behavior. The article also covers the connection between diet and mental health, noting that an increase in mental illness rates has been connected to troubled or single-parent households, difficulties in getting or keeping jobs, and other factors that may have an impact on crime. According to the study, the type of food eaten and the energy density of the diet may have an effect on calorie intake and the neuronal circuits in the brain, causing withdrawal symptoms that resemble addiction. In general, this work sheds light on the intricate connection between food, brain function, and behavior. It also discusses the effects of food insecurity on physical and mental health, as well as its probable connection to violent crime. The study makes the case for the necessity for further investigation into the relationship between food insecurity and violent crime in developed nations as well as the effects of nutrition on brain health and societal behavior (Figure 4A).

### 4.8. Gut–Brain Axis Communications and Behavioral Responses

The intestinal flora plays a crucial role in the gut–brain axis response pathway, regulating hormones, appetite, and energy metabolism (Table 1 and Figure 4B). Reward pathways in the brain play a crucial role in gut–brain communications, but their mechanisms remain unclear. Intestinal flora-derived metabolites influence leptin, ghrelin, and insulin, with the paraventricular nucleus and ventromedial prefrontal cortex playing essential roles. The vagus nerve and mitochondria modulate neurotransmitters and neurotransmission in gut–brain communications. The composition of intestinal flora significantly impacts sensory information and reward signaling in food selection, potentially preventing and treating obesity [56]. The previous study attempted to investigate the relationships between self-directed dieting, wellbeing measurements, and gut–brain axis processes that influence diet adherence via appetite mood, and metabolic control. The findings provided psychological and biological information on the effects of self-directed dieting, informed a framework for dieting safety, and established the possibility of biomarkers for risk management and enhancement of diet-based lifestyle interventions [57]. Herbal extracts and isolated substances have been shown in models of mood disorders to regulate gut microbiome structure and enhance depression-related behaviors. This demonstrates the study gap in natural product and gut microbiota interactions in mood disorders. Natural products’ therapeutic qualities could be explained by altering the microbiota–gut–brain axis [13]. Dietary flavonoids may have neuroprotective effects through direct and indirect mechanisms. They accumulate within the central nervous system, counteracting reactive oxygen species and promoting neuronal survival and proliferation. Flavonoids may also regulate brain function and host behavior by influencing gut microbiota composition and influencing the microbiota–gut–brain axis, potentially improving brain health and subsequently positive psychological behaviors [14]. The vagus nerve acts as a vital metabolic signal relay between the brain and the abdominal viscera. The effects of gut-based vagus nerve transmission on higher-order cognition domains, such as anxiety, depression, motivation for rewards, learning, and memory, have recently been discovered in both rodent models and humans. Consuming food activates vagal afferent transmission that originates in the gastrointestinal system, reducing anxiety and depression symptoms while enhancing motivation and memory. This procedure encourages the storage of information related to meals in the memory, making subsequent foraging activities easier. The topic of vagal tone regulation is also covered in relation to pathological illnesses including transcutaneous vagus nerve stimulation for phobias, major depressive disorders, and memory problems linked to dementia. These results demonstrate the significance of gastric vagus nerve signals in controlling neurocognitive functions and shaping adaptive behavioral responses [92].

This review explores the gut–brain axis, a bidirectional communication system between the gastrointestinal tract and the central nervous system. It highlights the role of intestinal flora, reward pathways, neurotransmitters, and the vagus nerve in mediating communication between the gut and the brain. The gut microbiota plays a crucial role in hormone regulation and energy metabolism, while the vagus nerve modulates neurotransmitters and neurotransmission. The vagus nerve, a major cranial nerve, acts as a relay between the brain and the abdominal viscera, reducing anxiety and depressive symptoms while enhancing motivation and memory. Natural products, such as herbal extracts and dietary flavonoids, have potential therapeutic effects on the gut microbiota and mood disorders. Understanding these mechanisms can inform the development of interventions for conditions related to appetite, mood, metabolic control, and cognitive function (Figure 4B).

### 4.9. The Role of Halal (Lawful) and Tayyib (Clean) Foods in the Mental Health of People

The highest standards of food hygiene in the Quran, Halal and Tayyib, are two fundamental notions in Islamic dietary regulations, and they have a substantial impact on people’s mental health (Table 1 and Figure 4C). Consuming Halal and Tayyib meals is thought to boost not only physical health but also spiritual well-being, which can have a favorable impact on mental health. Halal foods are those that are permitted under Islamic dietary requirements, whereas Tayyib foods are not only permissible but also clean and healthful. Shia teachings highlight the value of eating Halal and Tayyib meals, which are said to be more pure and beneficial for the body and soul. Consuming Halal and Tayyib meals can improve mental health in a variety of ways. For starters, it can help us feel more connected to God and His creation by promoting a sense of inner calm and tranquility. Second, eating Halal and Tayyib foods demands us to be attentive of what we eat and how it is cooked, which can build a sense of discipline and self-control. Furthermore, eating Halal and Tayyib foods can improve our physical health, which in turn can improve our mental health. A diet high in fruits, vegetables, and lean proteins, for example, can help lower inflammation in the body and promote excellent gut health, which has been associated with improved mental health outcomes. The Quran specifies the most ideal food hygiene and safety criteria as lawful Halal and Tayyib in order to meet all aspects of quality in terms of health and safety, nutritious, nourishing, and being legitimate needs. The implementation of such progressive standards is dependent on a correct interpretation of the Quran, the use of quality assurance systems, and the transition to organic manufacturing. Tayyib and Halal standards are quickly becoming household names around the world. The implementation of these standards provides both a very high added value to the country in terms of the production and supply of safe, healthy, and high-quality food, as well as high-quality human capital development, such as reforming faith, beliefs, thoughts, and behaviors, developing talents, boosting capabilities, providing comprehensive human health, and bringing humans closer to God [58,59].

According to Islamic dietary laws, the Quran addresses how Halal and Tayyib foods might promote mental wellness. According to this study, eating Halal and Tayyib meals can improve mental health by encouraging inner peace, calmness, and a sense of connectedness with God and His creation. Consuming Halal and Tayyib meals can also promote self-control and discipline, which can enhance mental health outcomes. In order to meet all aspects of quality in terms of health and safety, nutrition, nourishment, and being legitimate needs, this paper emphasizes the necessity of applying high standards of food cleanliness and safety criteria, as described in the Quran. In terms of the production and supply of safe, wholesome, and high-quality food as well as the development of human capital, such as changing one’s faith, beliefs, thoughts, and behaviors, cultivating one’s talents, enhancing one’s capabilities, providing for one’s overall health, and elevating one’s relationship with God, the implementation of Halal and Tayyib standards can offer a significant added value to the nation. According to the study, eating a diet rich in fruits, vegetables, and lean proteins can enhance gut health and reduce inflammation, both of which have been linked to better mental health outcomes. In order to apply these standards, this article emphasizes the significance of a proper interpretation of the Quran, the implementation of quality assurance methods, and the switch to organic manufacturing. Overall, the work sheds light on the possible contribution of Tayyib and Halal foods to mental health and the development of inner peace and quiet. According to the study, implementing high standards for food cleanliness and safety can benefit the nation significantly in terms of the production and supply of nutritious, safe, and high-quality food as well as the development of its human capital.

One of the limitations of this research that was not investigated was the topic regarding foods that are forbidden in different religions. Many religions around the world have halal and haram foods, which refer to the permissibility and prohibition of specific foods. The concept of halal and haram meals is very significant in Islam, and Muslims are expected to consume halal foods while avoiding haram and questionable foods. Haram foods in Islam include pork, alcohol, animal gelatin, alcohol-containing additives, and any form of some shellfish that is not considered allowed. The notion of kosher foods, known as kashrut, is accepted as dietary rule in Judaism. Meat murdered by a ritual slaughterer, some varieties of farmed fish with fins and scales, and mineral-derived sweeteners are all permitted foods. Haram foods include pork, shellfish, fish that are not regularly consumed and/or have health concerns, and dairy products containing meat. In Christianity, there is no distinction between halal and haram foods. However, many Christians abstain from eating pork for religious and/or health reasons. There are no specific foods that are considered haram in Christianity, although Christians generally avoid eating items that are known to be damaging to their health (Figure 4C).

## 5. Concluding Remarks and Future Prospects

**Figure 5:**
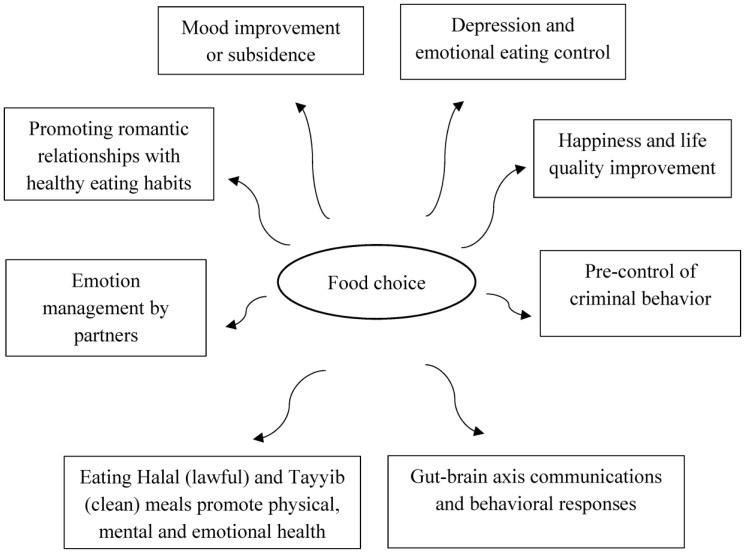
The general schematic diagram of the relationship between the type of food and the behavioral responses studied in this research.

The present search strategy and inclusion criteria were designed to ensure that the researchers identified all relevant articles while minimizing the risk of bias (Figure 5). In summary, choosing certain foods can make you feel better or worse about yourself. Emotion also has a significant impact on one’s appetite, dietary preferences, and desire to consume. A positive mood is influenced by wholesome foods including fruits, vegetables, nuts, and proteins as well as by drinking enough water and having very small amounts of coffee. Although the effect is only transitory and can have a harmful impact on one’s health, excessive consumption of fast food, sweets, and low-nutrient meals can also contribute to a pleasant mood. However, these unhealthy eating habits increase the risk of many chronic diseases. However, stressed individuals may show a strong urge to eat unhealthy foods to lift their spirits despite being aware that doing so would be detrimental to their mood. Our study also has some ramifications that could assist consumers in making wiser food decisions and leading healthier lives. For instance, couples should be conscious of their underlying relationship goals when they go out to eat. Women should be aware that their temptation will be to adopt their male partners’ eating habits when they first try to start a love connection. Females should be assisted in maintaining a healthy diet by either selecting a partner who eats well or intentionally fighting underlying inclinations by eating properly as a way of attractiveness enhancement. It is worth noting that some men prefer a nonconformist attitude when starting partnerships. As a result, females may not only keep a healthy diet through avoiding influence from poor eating partners, but they may also potentially increase their partner’s interest in a relationship by not adopting their male counterparts’ eating habits.

The current study also suggests that emotion suppressing may be a valid moderator of the association between mood worsening and consumption of food during artificially stressful marital conversations. This effect varied according to BMI; people with higher BMIs ate more when their moods become worse than people with lower BMIs. The findings also demonstrate how the partner’s suppression of emotions contributes to the relationship between mood and food consumption. It appears that, during a difficult conversation, a person’s food intake is related to both how that person manages his or her own emotions as well as how that person’s partner manages their own emotions. The physical and social worlds connect through nutrition, which can be thought of as the hardware and software of life as both are necessary for social behavior. The dietary approach to behavior is straightforward, compassionate, and obviously very effective. It enhances already-in-place offender programs. It is probably very cost-effective, and the only potential downside is improved health. It is a very promising strategy that has drawn the support of the Wellcome Trust, one of the top funding organizations in the world. Natural products like herbal extracts and flavonoids have potential therapeutic effects on gut microbiota and mood disorders. Understanding gut–brain axis communications can inform interventions for appetite, mood, metabolic control, and cognitive function. According to Shia teachings, eating Halal and Tayyib meals promotes physical, spiritual, and mental well-being. They may generate a sense of inner calm, discipline, and well-being that can enhance their mental health by being attentive of what they eat and attempting to ingest pure and healthful foods (Figure 5). The researchers hope to enhance the transparency and usefulness of their review for other researchers and readers in this field. This information will allow readers to assess the validity of the findings and replicate this search strategy for future research.

Future research could look into the effects of individual nutrients or dietary patterns on mental health outcomes. For example, studies may be conducted to investigate the effects of omega-3 fatty acids on depression or the benefits of a Mediterranean diet on anxiety. Furthermore, additional research is required to understand the mechanisms underlying the link between diet and mental health, such as the gut–brain axis.

## Figures and Tables

**Table 1:** Effect of food choice on psychological reactions and behavioral responses.

Objective	Key Sections	References
Food and Mood	Stress and depression may cause emotional eating	[18,19,20]
	Unhealthy food choices reduce satisfaction and psychosomatic benefits	[20,21,22]
	Consuming fruits and vegetables reduces stress levels	[19,23,24]
	Food’s antioxidants boost mood, reduce tension, and protect against anxiety	[25,26,27]
Food and Depression	Poor eating, smoking, and an inactive lifestyle raise the risk of depression	[4,5,28]
	Exclusion of certain foods increases depression risk	[29,30]
	Vitamin B complex, D, C, zinc, omega-3 fatty acids, and antioxidant deficiencies can negatively impact mood and potentially lead to depression. Nutrients like fruits, vegetables, and dairy are essential	[27]
	Consuming pro-inflammatory foods increases depression risk; low-inflammatory diets reduce symptoms	[31]
	Hormones like adrenaline, cortisol, and glucagon affect anxiety, hunger, and hypoglycemia	[32]
Food and Happiness	Research supports the idea that good dietary choices, like fruit and vegetable consumption, benefit physical and mental health	[20,33,34,35]
Romantic Relationships and Food	Couples eat over half of their meals together, strengthening and preserving relationships. Examining food preferences in romantic relationships can reveal unhealthy eating habits and potential obesity. Male eating habits impact females when relationship creation goals are active, while female eating patterns impact males when relationship maintenance goals are active.	[36,37,38,39]
Emotion suppression in romantic relationships and food consumption	Overweight women consume more on emotion-suppression days than non-obese women	[28,40,41]
	Thematic templates reveal three themes: social environment, social economics, and food nostalgia, focusing on cultural, familial, and economic factors impacting mood	[42,43,44,45,46,47,48,49,50,51,52]
Food Insecurity effect on Violent Crime	Food insecurity increases violence and civil strife in developing countries	[53,54,55]
	Fish, micronutrient-rich diet, and good nutrition reduce violent behavior rates	[55]
Gut–brain axis communications and behavioral responses	Herbal extracts regulate gut microbiome structure, enhance depression-related behaviors	[13,56,57]
	Dietary flavonoids influence gut microbiota composition and affect the vagus nerve, which controls neurocognitive functions and adaptive behavioral responses	[14,56,57]
Halal and Tayyib foods and mental health	Halal and Tayyib meals improve physical, spiritual, and mental health	[58,59]

## Data Availability

The data that support the findings of this study are available from the corresponding author upon reasonable request.
